# Mechanisms underlying extensive Ser129-phosphorylation in α-synuclein aggregates

**DOI:** 10.1186/s40478-017-0452-6

**Published:** 2017-06-15

**Authors:** Shigeki Arawaka, Hiroyasu Sato, Asuka Sasaki, Shingo Koyama, Takeo Kato

**Affiliations:** 1 Department of Neurology, Hematology, Metabolism, Endocrinology and Diabetology, Yamagata University Faculty of Medicine, 2-2-2 Iida-nishi, Yamagata, 990-9585 Japan; 2 Present address: Department of Internal Medicine IV, Osaka Medical College, 2-7 Daigaku-machi, Takatsuki, Osaka, 569-8686 Japan; 3 Present address: Laboratory of Research Resources, National Institute for Longevity Sciences, National Center for Geriatrics and Gerontology, 7-430, Morioka-cho, Obu, Aichi 474-8511 Japan

**Keywords:** Parkinson’s disease, α–Synuclein, Phosphorylation, Aggregation, Mitochondrial impairment, Proteasome pathway

## Abstract

Parkinson’s disease (PD) is characterized neuropathologically by intracellular aggregates of fibrillar α-synuclein, termed Lewy bodies (LBs). Approximately 90% of α-synuclein deposited as LBs is phosphorylated at Ser129 in brains with PD. In contrast, only 4% of total α-synuclein is phosphorylated at Ser129 in brains with normal individuals. It is unclear why extensive phosphorylation occurs in the pathological process of PD. To address this issue, we investigated a mechanism and role of Ser129-phosphorylation in regulating accumulation of α-synuclein. In CHO cells, the levels of Ser129-phosphorylated soluble α-synuclein were maintained constantly to those of total α-synuclein in intracellular and extracellular spaces. In SH-SY5Y cells and rat primary cortical neurons, mitochondrial impairment by rotenone or MPP^+^ enhanced Ser129-phosphorylation through increased influx of extracellular Ca^2+^. This elevation was suppressively controlled by targeting Ser129-phosphorylated α-synuclein to the proteasome pathway. Rotenone-induced insoluble α-synuclein was also targeted by Ser129-phosphoryation to the proteasome pathway. Experiments with epoxomicin and chloroquine showed that proteasomal targeting of insoluble Ser129-phosphorylated α-synuclein was enhanced under lysosome inhibition and it reduced accumulation of insoluble total α-synuclein. However, in a rat AAV-mediated α-synuclein overexpression model, there was no difference in the number of total α-synuclein aggregates between A53T mutant and A53T plus S129A double mutant α-synuclein, although Ser129-phosphorylated α-synuclein-positive aggregates were increased in rats expressing A53T α-synuclein. These findings suggest that Ser129-phosphorylation occurs against stress conditions, which increases influx of extracellular Ca^2+^, and it prevents accumulation of insoluble α-synuclein by evoking proteasomal clearance complementary to lysosomal one. However, Ser129-phosphorylation may provide an ineffective signal for degradation-resistant aggregates, causing extensive phosphorylation in aggregates.

**Electronic supplementary material:**

The online version of this article (doi:10.1186/s40478-017-0452-6) contains supplementary material, which is available to authorized users.

## Introduction

Although Parkinson’s disease (PD) is the most common movement disorder, there is currently no treatment for slowing or stopping disease progression. Identification of a promising target for PD therapies needs to elucidate how nigral dopaminergic neurons are lost and how Lewy bodies (LBs) and Lewy neurites are formed in surviving neurons, because these are key features of PD [6, 10]. LBs and Lewy neurites are aggregates of fibrillar α-synuclein (α-syn) [23]. As a characteristic point of LBs, approximately 90% of α-syn deposited in LBs is extensively phosphorylated at Ser129 [1, 7]. In sharp contrast, only 4% or less of total α-syn is phosphorylated at this residue in brains from individuals without PD [1, 7]. This disparity suggests that the levels of Ser129-phosphorylated α-syn are tightly regulated under physiological conditions, and extensive Ser129-phosphorylation occurs in conjunction with LB formation and dopaminergic neurodegeneration in PD. In a *Drosophila* model of PD, co-expression of α-syn and *Drosophila* G-protein-coupled receptor kinase 2 (Gprk2) was shown to generate Ser129-phosphorylated α-syn and enhance α-syn toxicity [4]. In a rat recombinant adeno-associated virus (rAAV)-based model, co-expression of A53T α-syn and human G-protein-coupled receptor kinase 6 (GRK6) accelerated α-syn-induced degeneration of dopaminergic neurons [18]. Conversely, the co-expression of wild-type α-syn and polo-like kinase 2 (PLK2) attenuated a loss of dopaminergic neurons [14]. Although the effect of Ser129-phosphorylation is still under debate, these findings show that Ser129-phosphorylation modulates α-syn toxicity. One simple explanation that links extensive α-syn phosphorylation in LBs with neurodegeneration is that Ser129-phosphorylation enhances α-syn aggregation and exerts a toxic or protective effect against neuronal damage. However, most in vitro studies have shown that Ser129-phosphorylation has no accelerating effect on the fibril formation of α-syn. The mechanism of extensive phosphorylation of α-syn in LBs remains unclear.

To address the issue, we first assessed how levels of phosphorylated α-syn are maintained in intra- and extracellular spaces using Chinese hamster ovary (CHO) cells. We then investigated how external stimulants affect α-syn phosphorylation in SH-SY5Y cells and primary rat cortical neurons. As external stimulants, we focused on intracellular Ca^2+^ and mitochondrial impairment, because previous studies demonstrated that α-syn phosphorylation by GRK5 is activated by Ca^2+^ and calmodulin (CaM), and α-syn can bind to CaM in a calcium-dependent manner [13, 15]. Mitochondrial complex I inhibition via administration of MPTP (1-methyl-4-phenyl-1,2,3,6-tetrahydropyridine) or rotenone causes a loss of dopaminergic neurons, and mitochondrial complex I dysfunction is found in PD patients, indicating the involvement of mitochondrial impairment in dopaminergic neurodegeneration of PD [11, 19, 21]. We also focused on the proteasome pathway as the competitive machinery, because soluble Ser129-phosphorylated α-syn was shown to degrade by the proteasome pathway [12]. The present study describes the regulation and dysregulation mechanisms of Ser129-phosphorylaion as an interplay among calcium, mitochondrial impairment, and proteasome clearance.

## Materials and methods

### Plasmid cDNA and reagents

Human wild-type α-syn cDNA was subcloned into the pcDNA3.1(+) vector (Thermo Fisher Scientific). Reagents were purchased from Sigma unless otherwise stated.

### Cell lines, rat primary cortical neuron cultures, and transfection

Human dopaminergic neuroblastoma SH-SY5Y cells (ECACC #94030304) were maintained in Ham’s F-12/Eagle’s minimum essential medium supplemented with 15% fetal bovine serum (FBS, Thermo Fisher Scientific), 2 mM l-glutamine (Thermo Fisher Scientific) and 1 × non-essential amino acids. SH-SY5Y cell lines stably expressing wild-type α-syn (wt-aS/SH #4) and Ser129-phosphorylation-incompetent S129A mutant α-syn (S129A-aS/SH #10) were used as described previously [8]. CHO cells were maintained in Ham’s F-12 supplemented with 10% FBS. Primary cortical neuron cultures were prepared from Sprague-Dawley rats. Neurons were isolated from the neocortex of embryonic day 18 rats and dissociated cells were plated at a density of 1 × 10^6^ cells on poly-D-lysine-coated 6-well plates. Neurons were maintained in serum-free neurobasal medium supplemented with B27 and GlutaMAX (Thermo Fisher Scientific) [12]. At intervals of 2 days, half of the plating medium was renewed. At 21 days in vitro (DIV), neurons were harvested for experiments. For transient transfection, cells were transfected with cDNA by using LipofectAMINE Plus reagent (Thermo Fisher Scientific) according to the manufacture’s protocol. The cells were harvested at 48 h post-transfection.

### Chemical treatments

To assess effects of intracellular Ca^2+^, at 16 h after plating wt-aS/SH cells onto 6-well plates, we checked the cells to be ∼80% confluent, and then the cells were incubated in fresh medium containing the indicated concentrations of calcium ionophore A23187 with or without the indicated concentrations of Ca^2+^ chelators (EGTA or BAPTA-AM) or CaM inhibitors (W-7 or calmidazolium chloride). In rat primary cortical neurons, they were cultured for 21 DIV and then incubated in fresh medium containing the indicated concentrations of A23187 with or without the indicated concentrations of EGTA, BAPTA-AM, or W-7. For mitochondrial complex I inhibition, at 16 h after plating parental SH-SY5Y cells or wt-aS/SH cells onto 6-well plates, we checked the cells to be ∼80% confluent, and then the cells were incubated in fresh medium containing the indicated concentrations of MPP^+^ iodide or rotenone with or without the indicated concentrations of EGTA, BAPTA-AM, or W-7. At 21 DIV, rat primary cortical neurons were also treated with the indicated concentrations of rotenone with or without EGTA, BAPTA-AM, or W-7. As a vehicle control, cells were treated with the same concentration of DMSO, which was used for dissolving chemicals other than EGTA.

To assess the metabolic fates of proteins in the cells, we performed experiments using the de novo protein synthesis inhibitor cycloheximide (CHX) [12]. At 16 h post-plating wt-aS/SH cells onto 6-well plates, we confirmed the cells to be ∼80% confluent. The cells were incubated in fresh medium containing 100 μM CHX for the indicated times. To test the effect of mitochondrial impairment and inhibition of the proteasome pathway on the metabolic fates of target proteins, we pre-incubated the cells with 10 μM rotenone or 0.1% DMSO for 8 h and treated them with CHX in the presence or absence of 10 μM MG132 for the indicated times.

To assess the relationship between the proteasome and lysosome pathways on the expression of α-syn, we treated cells with lysosome inhibitor chloroquine in the absence or presence of proteasome inhibitor epoxomicin (Peptide Institute Inc., Japan). At 16 h post-plating wt-aS/SH cells onto 6-well plates, we confirmed the cells to be ∼80% confluent. The cells were incubated in fresh medium containing 100 μM chloroquine with or without 100 nM epoxomicin for 24 h for the indicated times.

### Preparation of protein extracts and conditioned media

For preparation of cell lysates, cultured cells were suspended in buffer A (20 mM Tris-HCl, pH 7.4, 150 mM NaCl, 1% Triton X-100, 10% glycerol, 1 × protease inhibitor cocktail [Roche Diagnostic], 1 mM EDTA, 5 mM NaF, 1 mM Na_3_VO_4_, 1 × phosSTOP [Roche Diagnostic]), sonicated at 30 W for 1 s 5 times, and kept on ice for 30 min. After centrifugation at 12,000×g for 30 min, the resulting supernatant was collected and stored at −80 °C until use. Protein concentrations were measured by the BCA assay (Thermo Fisher Scientific).

For preparation of the conditioned media (CM), cells were plated onto 10 cm dishes. When cells were 90% confluent, we exchanged the growth media with 6 mL of Opti-MEM (Thermo Fisher Scientific). After further 24 h incubation, CM was collected and centrifuged at 6,000×g for 5 min to remove cell debris. Immediately, 6 mL of CM was added with 1/4 volume of 100% trichloroacetic acid (TCA), incubated for 30 min on ice, and centrifuged at 14,000×g for 5 min. The resultant pellet was washed three times with 300 μL of cold acetone, air dried, and dissolved in 100 μL of Laemmli’s sample buffer containing 2.5% β-mercaptoethanol.

For analyzing the levels of insoluble α-syn, 1% Triton X-100 cell lysates were separated by centrifugation at 100,000×g for 30 min [18]. Supernatants were collected as soluble fractions. Resultant pellets were washed with buffer A by centrifugation at 100,000×g for 30 min. The pellets were added with the solution containing 8 M urea and 2% SDS, sonicated at 30 W for 1 s 10 times, and then centrifuged at 100,000×g for 30 min. Resultant supernatants were used as insoluble fractions.

### Western blotting

Samples were denatured at 95 °C for 5 min in Laemmli’s sample buffer containing 2.5% β- mercaptoethanol. Equal protein amounts of denatured samples were subjected to SDS-PAGE on 13.5% polyacrylamide gels and then transferred to PVDF membranes (Immobilon-P, Millipore). The transferred membrane was incubated in phosphate-buffered saline (PBS) (10 mM phosphate, 137 mM NaCl, 2.7 mM KCl) containing 4% paraformaldehyde (PFA) with 0.1% glutaraldehyde for detecting Ser129-phosphorylated α-syn for 30 to 60 min or without glutaraldehyde for detecting other proteins as described previously [17]. After incubation, the membrane was washed in Tris-buffered saline (TBS, 25 mM Tris-HCl, pH 7.4, 137 mM NaCl, 2.7 mM KCl) containing 0.05% (*v*/v) Tween 20 (TBS-T) for 10 min 3 times. The membrane was blocked by TBS-T containing 5% skim milk for 30 min, incubated in TBS-T containing 2.5% skim milk and primary antibody overnight in the cold room, and further incubated in the same buffer containing the corresponding secondary antibody overnight in the cold room. When we detected phosphorylated α-syn, 50 mM NaF was added to TBS-T containing skim milk. To visualize the signal, membranes were treated with ECL plus (Thermo Fisher Scientific) for detection of total α-syn, including non-phosphorylated and Ser129-phosphorylated forms, and phosphorylated α-syn. Other proteins were detected by supersignal West Pico chemiluminescent substrate (Thermo Fisher Scientific). Signals were recorded using a CCD camera, VersaDog 5000 (Bio-Rad). Levels of total α-syn and Ser129-phosphorylated α-syn were estimated by measuring band intensities with Quantity One software (Bio-Rad). For more quantitative estimation of the expression levels of total α-syn and Ser129-phosphorylated α-syn, we used purified recombinant α-syn proteins and Ser129-phosphorylated α-syn proteins as standards [8, 12]. A set of diluted standards was subjected to SDS-PAGE along with samples. After quantifying band intensities of samples, we corrected their relative intensities by plotting them on the standard curve. The following antibodies were used: anti-α-syn (Syn-1, mouse monoclonal, which recognizes total α-syn independently of Ser129-phosphorylation, BD Transduction Laboratories), anti-Ser129-phosphorylated α-syn (EP1536Y, rabbit monoclonal, Abcam), and anti-β-actin (AC-15, mouse monoclonal, Sigma).

### rAAV-based rat model of Parkinson’s disease

The experiments using rats had been approved by the Animal Subjects Committee of Yamagata University [18]. We used rats expressing familial PD-linked A53T mutant or A53T plus S129A double mutant α-syn by unilaterally injecting a rAAV2 vector in the rat substantia nigra. These rats were the same as ones reported in our previous paper [18]. Methods concerning rAAV particles preparation and immunohistochemistry were described in this paper [18]. We analyzed the brain sections that were already immunostained with anti-human α-syn (LB509, 1: 200; Zymed Laboratories) and anti-Ser129-phosphorylated α-syn antibodies (EYPSYN-01, 1: 200; courtesy of Eisai). Briefly, the brains fixed by 4 ~ 8% PFA/PBS were coronally sectioned on a freezing microtome at a thickness of 30 μm. Sections were collected in 10 series to be regularly spaced at intervals of 300 μm from each other. For counting the number of α-syn aggregates in the striatum, we analyzed the injected sides of 4 to 6 sections around bregma by the optical fractionator method using Stereo Investigator software (MicroBrightField) [2]. The region of interest was traced and sampled using an Olympus BX50 microscope at a magnification of 4× and 10×, respectively. By setting the x-y sampling grid size equal to the counting frame size (330 × 330 μm), we scanned the whole area of the striatum on the section. We counted the number of α-syn aggregates larger than 5 μm in diameter. The size of aggregates was judged by measuring the maximum diameter with a “quick measure circle” tool or a “grid indicator” tool (5 μm × 5 μm/ one square) in this software.

### Statistical analysis

We performed each experiment at least three times. Data are expressed as mean ± standard deviation and n represents the number of samples. Homogeneity of variances was checked with Levene’s test. If the variances were homogenous, comparisons were performed by one-way analysis of variance (ANOVA) with Bonferroni’s post hoc test. In case of nonhomogeneity of variances, comparisons were performed by Welch-ANOVA with Games-Howell post hoc test (SPSS version 17, IBM). *P* values <0.05 were considered statistically significant.

### Data availability

The authors declare that the main data supporting the findings of this study are available within the article and Additional files 1 and 2.

## Results

### Regulation of Ser129-phosphorylated α-syn in intra- and extracellular spaces

**Fig. 1:**
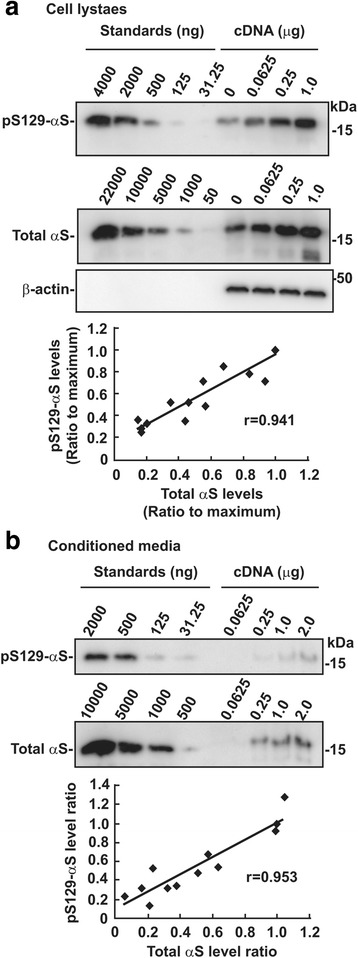
Relation of Ser129-phosphorylated α-syn levels to total α-syn ones in intra- and extracellular spaces. CHO cells were transfected with the empty vector or the indicated amounts of wild-type α-syn cDNA. Samples were loaded along with recombinant α-syn proteins and Ser129-phosphorylated α-syn proteins for standards, followed by western blotting. Bands of Ser129-phosphorylated α-syn and total α-syn, including phosphorylated and non-phosphorylated forms, were detected by EP1536Y and Syn-1 antibody, respectively. Relative band intensities of Ser129-phosphorylated α-syn and total α-syn were corrected by plotting them on the standard curves, and then normalized to the intensities of β-actin. **a** Relation in intracellular α-syn. Cell lystaes (10 μg/lane) were loaded. **b** Relation in extracellular α-syn. After TCA-precipitated proteins were resolved by Laemmli’s sample buffer, samples corresponding to 20% of CM volume were loaded. Graphs show the positive correlation between Ser-129 phosphorylated and total α-syn levels

We compared the levels of Ser129-phosphorylated α-syn with those of total α-syn, including non-phosphorylated and phosphorylated forms, by transfecting either an empty vector or three different amounts of α-syn cDNA into CHO cells. We used CHO cells in this experiment to achieve transient transfection with relatively high efficiency. In cell lysates, the levels of total or Ser129-phosphorylated α-syn elevated with increasing amounts of transfected α-syn cDNA (Fig. 1a). Expression of all samples was assessed, revealing that the levels of Ser129-phosphorylated α-syn positively correlated with those of total α-syn from endogenous proteins (*n* = 12, *r* ^2^ = 0.885) (Fig. 1a). The same correlation in levels between Ser129-phosphorylated α-syn and total α-syn was observed in conditioned media (CM) (*n* = 9, *r* ^2^ = 0.908), although endogenous α-syn was undetectable (Fig. 1b). These findings showed that Ser129-phosphorylation was modulated at a constant rate in proportion to levels of total α-syn, and this relationship was also observed in secreted α-syn.

### Effects of calcium on Ser129-phosphorylation of α-syn

**Fig. 2:**
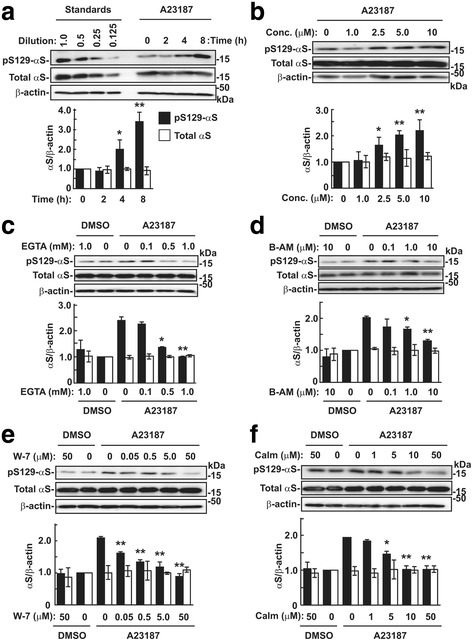
Effects of Ca^2+^ on Ser129-phosphorylation of α-syn. SH-SY5Y cell lines stably expressing wild-type α-syn (wt-aS/SH #4) were incubated in media containing 5 μM calcium ionophore A23187 except **b**. As vehicle control, cells were treated with DMSO at the same final concentration as reagents used. Cell lysates (15 μg/lane) were loaded on SDS-PAGE and analyzed by western botting with EP1536Y, Syn-1, or anti-β-actin (AC-15) antibody. **a** Effect of A23187 incubation time on Ser129-phosphorylation. Cells were treated with A23187 for the indicated time points until 8 h. **b** Effect of A23187 concentrations on Ser129-phosphorylation. Cells were treated with A23187 at the indicated concentrations for 8 h. **c, d** Effect of extracellular Ca^2+^ chelator EGTA (**c)** or intracellular Ca^2+^ chelator BAPTA-AM (B-AM) (**d**) on A23187-induced Ser129-phosphorylation. Cells were incubated in media containing 5 μM A23187 with the indicated concentrations of EGTA or BAPTA-AM for 4 h. **e, f** Effect of CaM inhibitor W-7 (**e**) or calmidazolium (Calm) (**f**) on A23187-induced Ser129-phosphorylation. Cells were incubated in media containing A23187 with the indicated concentrations of W-7 or calmidazolium for 4 h. Representative blots are shown. In the graphs of **a** to **f**, relative band intensities of Ser129-phosphorylated α-syn and total α-syn were normalized to those of β-actin. Data represent means ± SD and *P* values were estimated by one-way ANOVA with Bonferroni correction or Welch-ANOVA with Games-Howell post hoc test for unequal-variances (*, *P* < 0.05; **, *P* < 0.01)

To test the effect of intracellular Ca^2+^ on Ser129-phosphorylation of α-syn, we incubated SH-SY5Y cell lines, which stably expressed wild-type α-syn (wt-aS/SH #4) [8], for 8 h in media containing 5 μM calcium ionophore A23187. The levels of Ser129-phosphorylated α-syn significantly increased after 4 h incubation (1.99 ± 0.48-fold increase at 4-h incubation, *P* = 0.033; 3.40 ± 0.45-fold increase at 8-h incubation, *P* = 0.001, *n* = 5, each group), compared with vehicle control cells (Fig. 2a). Cells were then incubated with various concentrations of A23187 for 4 h. Phosphorylated α-syn levels significantly increased (1.64 ± 0.31-fold increase at 2.5 μM, *P* = 0.019; 2.03 ± 0.17-fold increase at 5.0 μM, *P* < 0.001; 2.20 ± 0.40-fold at 10 μM, *P* = 0.004, *n* = 6, each group) (Fig. 2b). However, the levels of total α-syn were not altered by A23187 (Fig. 2a and b). The A23187-mediated Ser129-phosphorylation of α-syn (2.39 ± 0.14-fold increase) was significantly inhibited by the addition of EGTA (1.36 ± 0.04-fold increase at 0.5 mM, *P* = 0.015; 1.00 ± 0.39-fold increase at 1.0 mM, *P* = 0.007, *n* = 3, each group) (Fig. 2c). Additionally, A23187-mediated Ser129-phosphorylation of α-syn (2.03 ± 0.04-fold increase) was significantly inhibited by adding BAPTA-AM (1.66 ± 0.07-fold increase at 1.0 μM, *P* = 0.015; 1.31 ± 0.05-fold increase at 10 μM, *P* < 0.001, *n* = 3, each group) (Fig. 2d). EGTA and BAPTA-AM have been shown to chelate extracellular and intracellular Ca^2+^, respectively [3, 9]. The present findings showed that A23187-mediated Ser129-phosphorylation was caused by raising intracellular Ca^2+^ concentrations from extracellular sources. A23187-mediated Ser129-phosphorylation of α-syn (2.09 ± 0.05-fold increase) was significantly blocked by the addition of CaM inhibitor W-7 from 0.05 μM (1.61 ± 0.05-fold increase, *P* = 0.002) and different CaM inhibitor calmidazolium chloride from 5.0 μM (1.47 ± 0.07-fold increase, *P* = 0.030, *n* = 3, each group), compared with vehicle control cells (Fig. 2e and f). These findings showed that CaM was involved in A23187-mediated Ser129-phosphorylation.

To test whether A23187 enhanced Ser129-phosphorylation of α-syn in neurons, we examined the effects of A23187 in rat primary cortical neurons. The levels of Ser129-phosphorylated α-syn significantly increased with 0.25 μM A23187 (2.58 ± 0.59-fold increase, *P* = 0.013, *n* = 5), compared with vehicle control cells (Additional file 1: Figure S1a). However, the levels of total α-syn remained unaltered. Unlike wt-aS/SH cells, the A23187 effect was attenuated at a higher concentration (Additional file 1: Figure S1A). A23187-mediated Ser129-phosphorylation of α-syn (1.78 ± 0.13-fold increase) was significantly inhibited by 0.5 mM EGTA (1.28 ± 0.17-fold increase, *P* = 0.023, *n* = 4, each group), and A23187-mediated Ser129-phosphorylation of α-syn (3.30 ± 0.28-fold increase) was significantly inhibited by BAPTA-AM (2.42 ± 0.31-fold increase at 0.5 μM, *P* = 0.010; 2.00 ± 0.20-fold increase at 1.0 μM, *P* < 0.001, *n* = 5, each group) (Additional file 1: Figure S1b and c). Additionally, A23187-mediated Ser129-phosphorylation of α-syn (1.56 ± 0.10-fold increase) was blocked by 20 μM W-7 (0.99 ± 0.20-fold increase, *P* = 0.021, *n* = 3, each group) (Additional file 1: Figure S1d). These findings showed that A23187 similarly enhanced Ser129-phosphorylation of α-syn via increased influx of extracellular Ca^2+^ and CaM in primary cortical neurons.

### Effects of mitochondrial complex I inhibition on Ser129-phosphorylation of α-syn

**Fig. 3:**
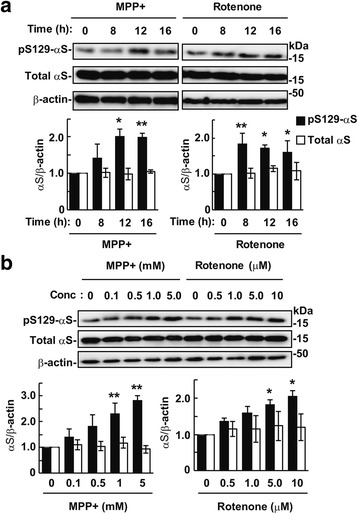
Effects of mitochondrial complex I inhibitors MPP^+^ and rotenone on Ser129-phosphorylation of α-syn. Cell lysates (10 μg/lane) were loaded on SDS-PAGE and analyzed by western botting with EP1536Y, Syn-1, or AC-15 antibody. **a** Effect of incubation time of MPP^+^ and rotenone on Ser129-phosphorylation. Wt-aS/SH #4 cells were incubated in media containing 1 mM MPP^+^ or 5 μM rotenone for the indicated time points until 16 h. Vehicle controls were treated with DMSO at the same final concentration as each reagent. **b** Effect of concentrations of MPP^+^ or rotenone on Ser129-phosphorylation. Cells were incubated in media containing the indicated amounts of MPP^+^ or rotenone for 12 h. Graphs show relative band intensities of Ser129-phosphorylated α-syn and total α-syn. They were normalized to those of β-actin. Data represent means ± SD and *P* values were estimated by one-way ANOVA with Bonferroni correction or Welch-ANOVA with Games-Howell post hoc test for unequal-variances (*, *P* < 0.05; **, *P* < 0.01)

To assess the effects of mitochondrial complex I inhibition on Ser129-phosphorylation of α-syn, we incubated wt-aS/SH cells in media containing either 1 mM MPP^+^ or 5 μM rotenone for 16 h. In MPP^+^-treated cells, the levels of Ser129-phosphorylated α-syn significantly increased and peaked after 12 h (2.01 ± 0.21-fold increase, *P* = 0.034, *n* = 3), compared with vehicle control cells (Fig. 3a). In rotenone-treated cells, Ser129-phosphorylated α-syn levels significantly increased and peaked after 8 h (1.84 ± 0.28-fold increase, *P* = 0.008, *n* = 3) (Fig. 3a). After incubation for 12 h, Ser129-phosphorylated α-syn levels significantly increased from 1 mM of MPP^+^ (2.30 ± 0.42-fold increase, *P* = 0.006, *n* = 3) or 5 μM of rotenone (1.82 ± 0.14-fold increase, *P* = 0.030, *n* = 3) in a dose-dependent manner (Fig. 3b). Total α-syn levels remained unchanged by MPP^+^ or rotenone (Fig. 3a and b).

**Fig. 4:**
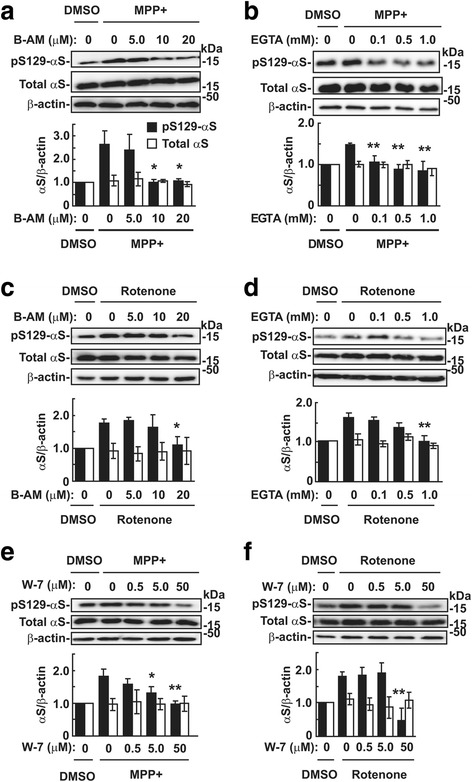
Role of Ca^2+^ and CaM in mitochondria complex I inhibitor-induced Ser129-phosphorylation of α-syn. As vehicle control, cells were treated with DMSO at the same final concentration as reagents used. Cell lysates (10 μg/lane) were loaded on SDS-PAGE and analyzed by western botting with EP1536Y, Syn-1, or AC-15 antibody. **a, b** Effect of intracellular Ca^2+^ chelator BAPTA-AM (**a**) or extracelluar Ca^2+^ chelator EGTA (**b**) on MPP^+^-induced Ser129-phosphorylation. Wt-aS/SH #4 cells were incubated in media containing 1 mM MPP^+^ and the indicated concentrations of BAPTA-AM or EGTA for 16 h. **c, d** Effect of BAPTA-AM (**c**) and EGTA (**d**) on rotenone-induced Ser129-phosphorylation. Wt-aS/SH #4 cells were incubated in media containing 5 μM rotenone and the indicated concentrations of BAPTA-AM or EGTA for 16 h. **e, f** Effect of CaM inhibitor W-7 on MPP^+^ (**e**)- or rotenone (**f**)-induced Ser129-phosphorylation. Cells were incubated in media containing 1 mM MPP^+^ or 5 μM rotenone with W-7 at the indicated concentrations for 16 h. Representative blots are shown. In the graphs of **a** to **f**, relative band intensities of Ser129-phosphorylated α-syn and total α-syn were normalized to those of β-actin. Data represent means ± SD and *P* values were estimated by one-way ANOVA with Bonferroni correction (*, *P* < 0.05; **, *P* < 0.01)

To identify the mechanism of mitochondrial complex I inhibition-mediated Ser129-phosphorylation of α-syn, we treated wt-aS/SH cells with 1 mM MPP^+^ in the presence or absence of Ca^2+^ chelators. MPP^+^-mediated Ser129-phosphorylation of α-syn (2.38 ± 0.96-fold increase,) was significantly inhibited by BAPTA-AM (1.08 ± 0.06-fold increase at 10 μM, *P* = 0.001; 1.04 ± 0.20-fold increase at 20 μM, *P* = 0.008, *n* = 3, each group) (Fig. 4a). To assess whether MPP^+^-mediated Ser129-phosphorylation of α-syn was due to Ca^2+^ leakage from damaged mitochondria, cells were treated with extracellular Ca^2+^ chelator EGTA. MPP^+^-mediated Ser129-phosphorylation of α-syn (1.49 ± 0.04-fold increase) was significantly inhibited by EGTA (1.06 ± 0.15-fold increase at 0.1 mM, *P* = 0.013; 0.89 ± 0.11-fold increase at 0.5 mM, *P* = 0.001; 0.85 ± 0.23-fold increase at 1.0 mM, *P* = 0.016, *n* = 5, each group) (Fig. 4b). Similarly, rotenone-mediated Ser129-phosphorylation of α-syn (1.83 ± 0.19-fold increase) was significantly inhibited by 20 μM BAPTA-AM (0.97 ± 0.18-fold increase, *P* = 0.001, *n* = 3, each group) (Fig. 4c). Rotenone-mediated Ser129-phosphorylation of α-syn (1.72 ± 0.08-fold increase, *n* = 3) was significantly inhibited by EGTA (1.19 ± 0.05-fold increase at 0.5 mM, *P* = 0.001; 1.08 ± 0.13-fold increase at 1.0 mM, *P* < 0.001, *n* = 3, each group) (Fig. 4d). Additionally, Ser129-phosphorylation of α-syn by MPP^+^ (2.04 ± 0.06-fold increase) was inhibited by W-7 (1.34 ± 0.10-fold increase at 5.0 μM, *P* = 0.025; 0.86 ± 0.09-fold increase at 50 μM, *P* < 0.001, *n* = 3, each group) (Fig. 4e). Ser129-phosphorylation of α-syn by rotenone (1.79 ± 0.08-fold increase) was inhibited by 50 μM W-7 (1.20 ± 0.06-fold increase, *P* < 0.001, *n* = 3, each group) (Fig. 4f). These findings showed that MPP^+^ and rotenone-mediated Ser129-phosphorylation of α-syn was the result of increased intracellular Ca^2+^ concentrations from extracellular sources, and CaM could modulate this effect.

To determine whether mitochondrial complex I inhibition enhances Ser129-phosphorylation of α-syn in neurons, we examined the effect of rotenone in rat primary cortical neurons. Ser129-phosphorylated α-syn levels significantly increased by rotenone treatment (2.31 ± 0.59-fold increase at 1.0 nM, *P* < 0.001; 1.68 ± 0.09-fold increase at 10 nM, *P* = 0.036, *n* = 4, each group), compared with vehicle control cells (Additional file 2: Figure S2a). The effect of rotenone on Ser129-phosphorylation peaked at 1.0 nM. However, total α-syn levels remained unchanged. The rotenone-mediated Ser129-phosphorylation of α-syn (1.68 ± 0.09-fold increase) was significantly inhibited by BAPTA-AM (1.16 ± 0.19-fold increase at 0.5 μM, *P* = 0.050; 0.80 ± 0.12-fold increase at 1.0 μM, *P* = 0.004, *n* = 5, each group). The rotenone-mediated Ser129-phosphorylation of α-syn (1.54 ± 0.11-fold increase) was significantly inhibited by 0.5 mM EGTA (1.17 ± 0.04-fold increase, *P* = 0.005, *n* = 5, each group) (Additional file 2: Figure S2b and c). Additionally, rotenone-mediated Ser129-phosphorylation of α-syn (1.89 ± 0.14-fold increase) was significantly inhibited by 20 μM W-7 (1.24 ± 0.15-fold increase, *P* = 0.020, *n* = 3, each group) (Additional file 2: Figure S2d). These findings showed that rotenone enhanced Ser129-phosphorylation of α-syn by increased influx of extracellular Ca^2+^ and CaM in primary cortical neurons.

### Role of the proteasome pathway in mitochondrial complex I inhibition-mediated Ser129-phosphorylation of α-syn

**Fig. 5:**
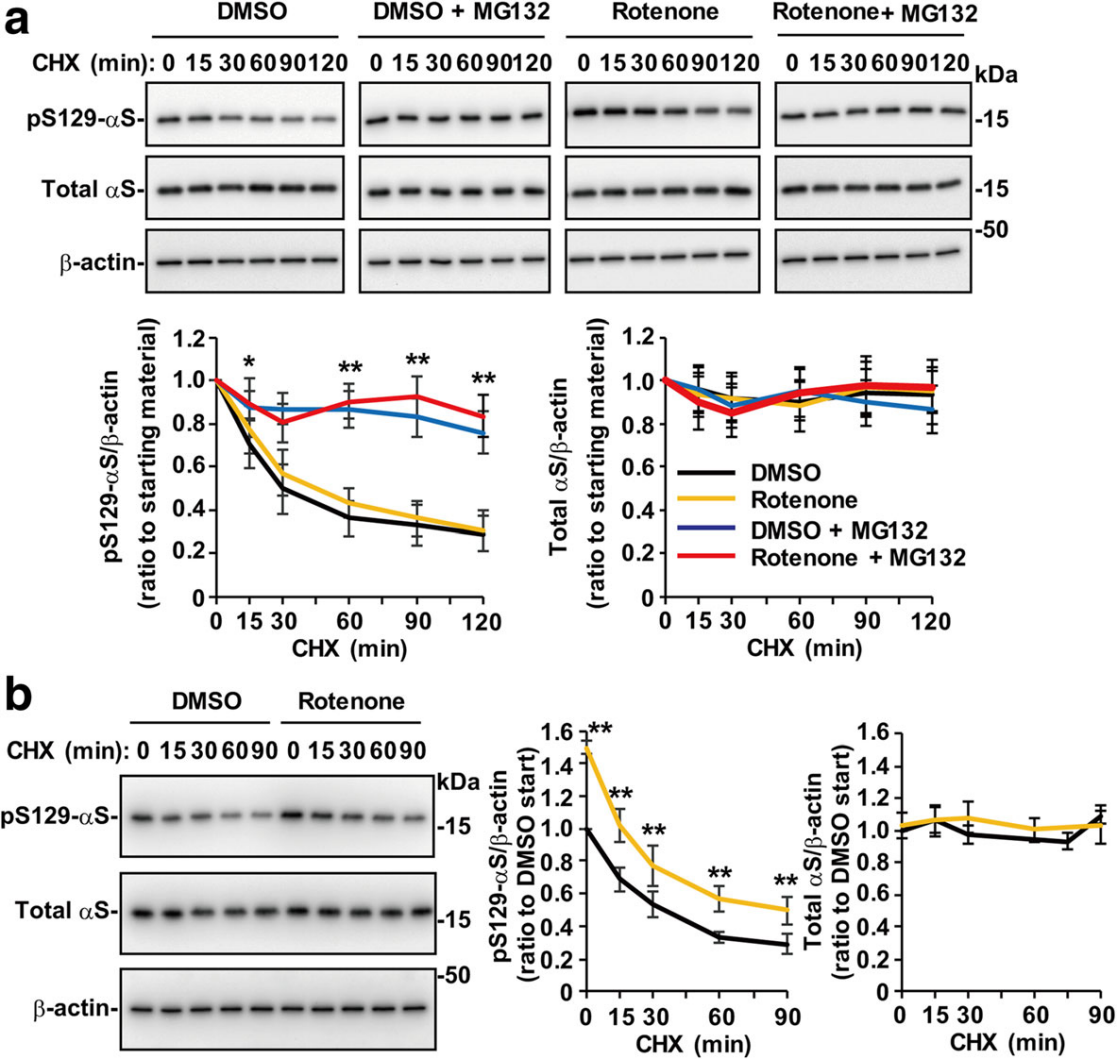
Ser129-mediated proteasomal targeting of soluble α-syn in mitochondrial complex I inhibition by rotenone. In each treatment, the concentration of DMSO was prepared to be equal. Cell lysates (2.5 μg/lane) were loaded on SDS-PAGE and analyzed by western botting with EP1536Y, Syn-1, or AC-15 antibody. **a** CHX-chase experiments for analyzing alteration in the metabolic fates of Ser129-phosphorylated and total α-syn by MG132 treatment. Wt-aS/SH #4 cells were pre-incubated in media containing either DMSO or 10 μM rotenone for 8 h. Then, they were incubated in fresh media further containing 100 μM cycloheximide (CHX) and/or 10 μM MG132 until 120 min. **b** Comparison of metabolic fates of rotenone-induced Ser129-phosphorylated α-syn with physiologically phosphorylated α-syn. Cells were pre-incubated in media containing DMSO or 10 μM rotenone for 8 h before CHX-chase experiments. Representative blots are shown. In **a** and **b**, graphs show the metabolic fates of Ser129-phosphorylated α-syn (*left*) and total α-syn (*right*). Black, yellow, blue and red lines represent treatment with DMSO, rotenone, MG132 and rotenone plus MG132, respectively. Data show means ± SD and *P* values were estimated by one-way ANOVA with Bonferroni correction (*, *P* < 0.05; **, *P* < 0.01)

To assess whether mitochondrial complex I inhibition enhances Ser129-phosphorylation of α-syn by impairing the degradation system, we investigated the metabolic fates of α-syn using cycloheximide (CHX)-chase experiments in wt-aS/SH cells. As previously reported, the levels of Ser129-phosphorylated α-syn rapidly decreased (Fig. 5a). When cells were pretreated with 10 μM rotenone for 8 h followed by CHX-chase experiments, Ser129-phosphorylated α-syn levels similarly decreased, although the starting levels were higher with rotenone treatment (Fig. 5a). This finding suggested that the rotenone-enhanced Ser129-phosphorylation was independent of degradation. To determine the degradation effect of rotenone-mediated Ser129-phosphorylation of α-syn, we performed CHX-chase experiments using cells treated with proteasome inhibitor MG132 in the absence or presence of rotenone. As previously reported, in the absence of rotenone, the rapid decrease in Ser129-phosphorylated α-syn was blocked (Fig. 5a). In the presence of rotenone, Ser129-phosphorylated α-syn attenuation was also blocked by MG132 (Fig. 5a). The levels of total α-syn remained unchanged in the CHX-chase experiments. To further compare the metabolic fates of α-syn in the absence or presence of rotenone, Ser129-phosphorylated α-syn levels were simultaneously assessed in vehicle control and rotenone-treated cell lysates on the same gel. The attenuation rate of Ser129-phosphorylated α-syn was comparable between vehicle control and rotenone-treated cells (Fig. 5b). These findings showed that rotenone did not impair the proteasome pathway under this experimental condition, and the rotenone-induced increase in Ser129-phosphorylation was suppressively controlled by pushing Ser129-phosphorylated α-syn along the proteasome pathway.

### Role of Ser129-phosphorylation in α-syn solubility change by mitochondrial complex I inhibition

**Fig. 6:**
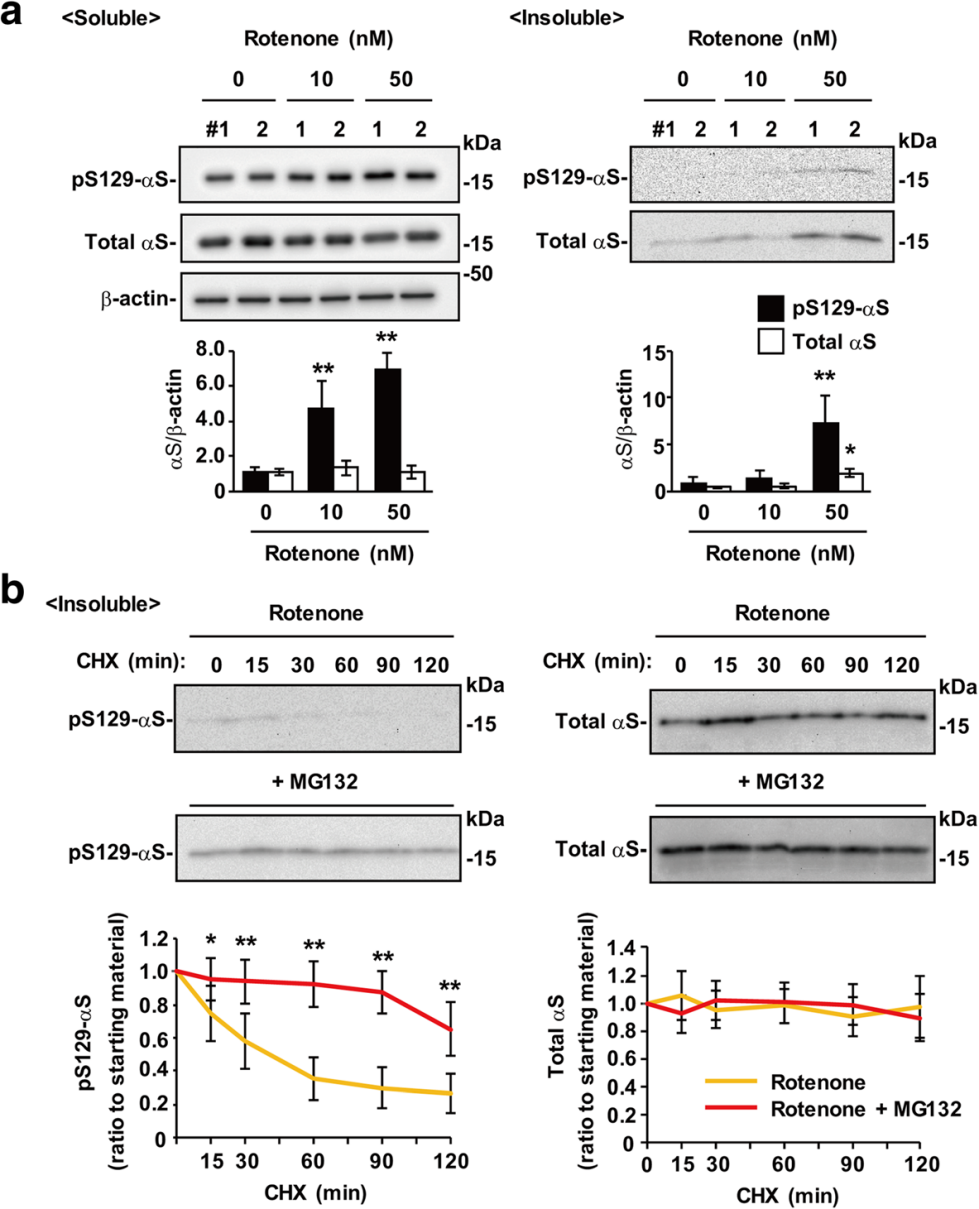
Effect of Ser129-phosphorylation on α-syn solubility change by mitochondrial complex I inhibition. Wt-aS/SH #4 cells were fractionated into 1% Triton X-100 soluble and insoluble fractions by centrifugation at 100,000×g for 30 min. 1% Triton X-100 insoluble pellets were resolved by 8 M urea / 2% SDS solution. In 1% Triton X-100 soluble fractions, the extract (2.5 μg / lane) were loaded onto SDS-PAGE. In 1% Triton X-100 insoluble fractions, samples corresponding to 15 μg of soluble fractions were loaded. These samples were analyzed by western botting with EP1536Y, Syn-1, or AC-15 antibody. **a** Solubility change of α-syn by rotenone treatment. Cells were incubated by 10 or 50 nM rotenone for 5 days. The representative blots are shown. **b** CHX-chase experiments for analyzing alteration in the metabolic fates of insoluble Ser129-phosphorylated and total α-syn by MG132 treatment. After treatment with 50 nM rotenone for 5 days, cells were incubated in fresh media further containing 100 μM cycloheximide (CHX) and either 0.1% DMSO or 10 μM MG132 until 120 min. Graphs show the metabolic fates of Ser129-phosphorylated α-syn (*left*) and total α-syn (*right*). Data show means ± SD and *P* values were estimated by one-way ANOVA with Bonferroni correction (*, *P* < 0.05; **, *P* < 0.01)

To determine the role of Ser129-phosphorylation in the accumulation of insoluble α-syn, we first examined whether detergent-insoluble α-syn proteins were present in rotenone-treated cells. Wt-aS/SH cells were incubated in low concentrations of rotenone (10 nM and 50 nM) for 5 days. The cells were then separated into 1% Triton X-100-soluble and 1% Triton X-100-insoluble fractions by centrifugation at 100,000×g for 30 min. The 1% Triton X-100-insoluble fractions were resolved in a solution containing 8 M urea / 2% SDS. Western blots of 1% Triton X-100-soluble fractions showed that 10 and 50 nM rotenone elevated Ser129-phosphorylated α-syn levels without altering total α-syn levels. When we analyzed 1% Triton X-100-insoluble fractions, 50 nM rotenone generated insoluble total α-syn (Fig. 6a). The levels of insoluble total α-syn increased by 3.89 ± 0.84-fold (*P* = 0.011, *n* = 4) as compared with vehicle control cells (Fig. 6a). Although the Ser129-phosphorylated α-syn signals were very faint, insoluble Ser129-phosphorylated α-syn levels also increased by 14.40 ± 6.23-fold (*P* = 0.004, *n* = 4) (Fig. 6a). We then performed CHX-chase experiments using cells pretreated with 50 nM rotenone for 5 days, followed by incubation in media containing DMSO or 10 nM MG132 for 8 h. In the absence of MG132, insoluble Ser129-phosphorylated α-syn levels rapidly attenuated before 120 min (Fig. 6b). MG132 significantly blocked this attenuation (Fig. 6b). However, insoluble total α-syn proteins remained unaltered during the observation time in the absence or presence of MG132 (Fig. 6b). These findings showed that Ser129-phosphorylation also pushed rotenone-induced insoluble α-syn through the proteasome pathway.

### Ser129-phosphorylation-mediated α-syn clearance in the proteasome and lysosome pathways

**Fig. 7:**
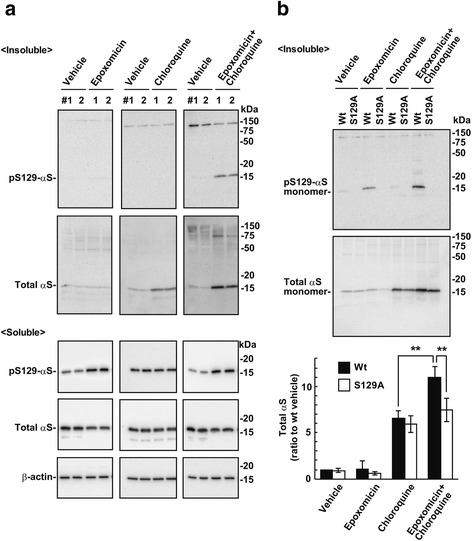
Relation of Ser129-phosphorylation-mediated α-syn clearance between the proteasome and lysosome pathways. Wt-aS/SH #4 cells were fractionated into 1% Triton X-100 soluble and insoluble fractions by centrifugation at 100,000×g for 30 min. In 1% Triton X-100 soluble fractions, the extract (2.5 μg / lane) were loaded onto SDS-PAGE. In 1% Triton X-100 insoluble fractions, samples corresponding to 15 μg of soluble fractions were loaded. Samples were analyzed by western botting with EP1536Y, Syn-1, or AC-15 antibody. **a** Effect of proteasomal and lysosomal inhibitions on the metabolism of α-syn. Cells were incubated by either 100 nM epoxomicin, 100 μM chloroquine, or 100 nM epoxomicin plus 100 μM chloroquine for 24 h. Vehicle control cells were treated with the same concentration of DMSO. Upper and lower panels show the blots of 1% Triton X-100 insoluble fractions and 1% Triton X-100 soluble fractions, respectively. **b** Effect of Ser129-phosphorylation on the metabolism of α-syn in proteasomal and lysosomal inhibitions. Wt-aS/SH #4 cells and S129A-aS/SH #10 cells were incubated by either 100 nM epoxomicin, 100 μM chloroquine, or epoxomicin plus chloroquine for 24 h. Upper panels show the blots of 1% Triton X-100 insoluble fractions. Graph shows relative ratios of total α-syn to vehicle control cells. Data represent means ± SD and *P* values were estimated by one-way ANOVA with Bonferroni correction (*, *P* < 0.05; **, *P* < 0.01)

To analyze Ser129-phosphorylation-mediated targeting of insoluble α-syn proteins in the degradation pathway, we examined the relationship between the proteasome and lysosome pathways. Wt-aS/SH cells were incubated in media containing 10 nM of selective proteasome inhibitor epoxomicin or 100 μM of chloroquine for 16 h. As shown in Fig. 7a (*left panels*), epoxomicin did not affect the levels of 1% Triton X-100-insoluble Ser129-phosphorylated α-syn, but did increase 1% Triton X-100-soluble Ser129-phosphorylated α-syn levels. Additionally, epoxomicin did not affect 1% Triton X-100-insoluble total α-syn levels. Chloroquine treatment failed to induce expression of insoluble Ser129-phosphorylated α-syn, but insoluble total α-syn did accumulate (Fig. 7a, *middle panels*). When cells were co-incubated in media containing epoxomicin and chloroquine, insoluble Ser129-phosphorylated α-syn proteins were generated in conjunction with the accumulation of insoluble total α-syn (Fig. 7a *right panels*). These findings showed that proteasomal targeting of insoluble Ser129-phosphorylated α-syn was more activated under lysosome inhibition. To further test the effect of Ser129-phosphorylation on the metabolism of insoluble α-syn proteins, we compared insoluble total α-syn levels between cells expressing wild-type α-syn (wt-aS/SH cells) and Ser129-phosphorylation incompetent S129A mutant α-syn (S129A-aS/SH cells). In the wt-aS/SH cells, epoxomicin and chloroquine treatment yielded insoluble total α-syn (11.00 ± 1.17-fold increase as compared with vehicle control cells, *n* = 5) more abundantly than chloroquine single treatment (6.51 ± 0.82-fold increase, *P* < 0.001, *n* = 5) (Fig. 7b). Additionally, epoxomicin and chloroquine treatment elevated insoluble total α-syn levels in wt-aS/SH more greatly than S129A-aS/SH cells (7.49 ± 1.27-fold increase, *P* < 0.001, *n* = 5) (Fig. 7b). S129A-aS/SH cells exhibited no Ser129-phosphorylated α-syn signals in the insoluble fractions (Fig. 7b). These findings showed that Ser129-phosphorylation prevented insoluble α-syn accumulation by evoking proteasomal clearance complementary to lysosomal clearance.

### Effect of Ser129-phosphorylation on α-syn aggregate formation in a rat AAV-mediated α-syn overexpression model

**Fig. 8:**
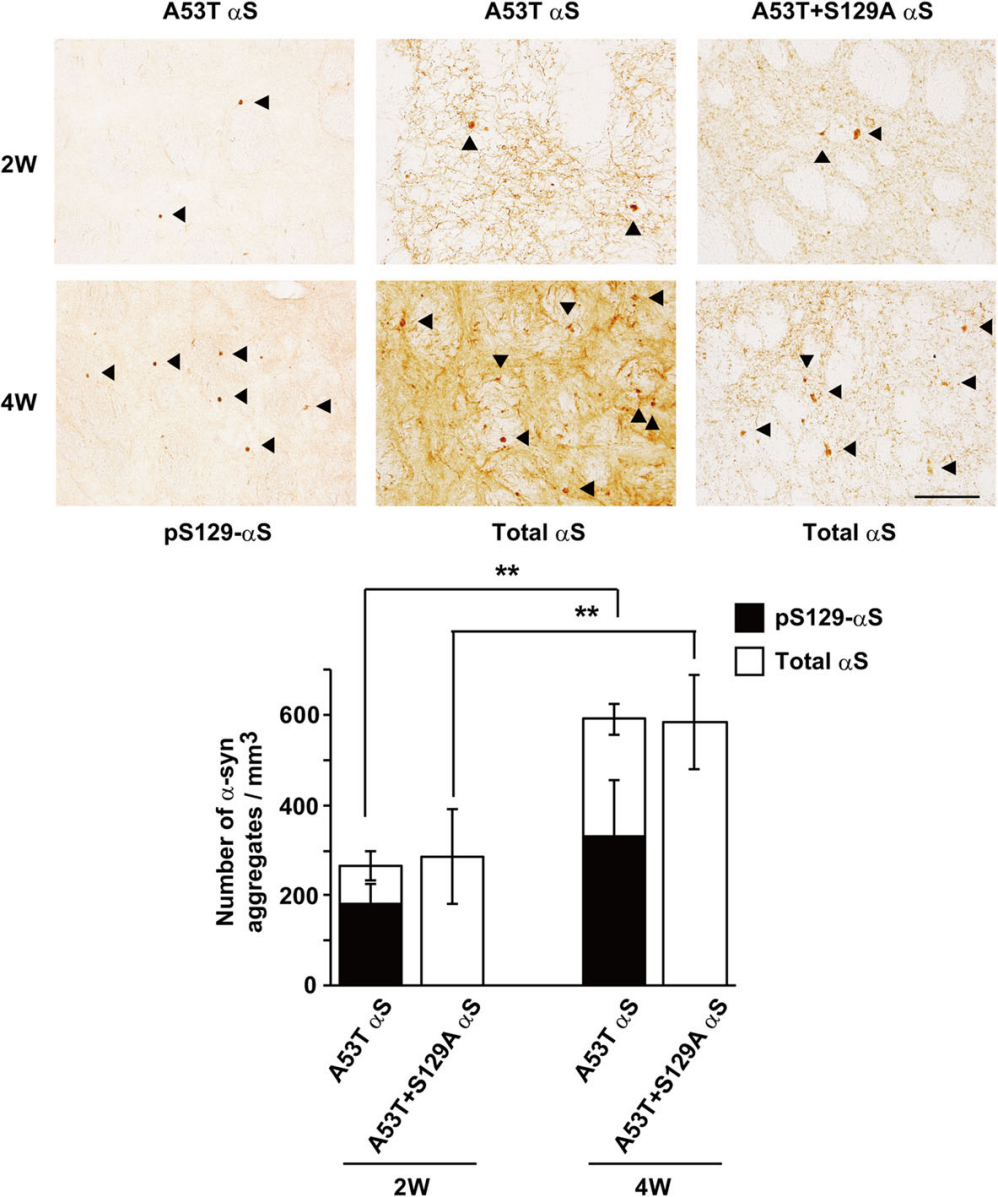
Effect of Ser129-phosphorylation on α-syn aggregate formation in a rat AAV-mediated α-syn overexpression model. Rats were sterotaxically injected with rAAV particles into the substantia nigra, and they expressed A53T mutant α-syn or A53T plus S129A double mutant α-syn. These rats were used in our previous study [18]. Information on the expression levels and toxicity is described in this paper [18]. Upper panels show the photomicrographs of rat striatum immunohistochemically stained with antibody specific to Ser129-phosphorylated α-syn and human total α-syn (LB509). We counted the number of α-syn-positive aggregates larger than 5 μm in diameter. Arrow heads indicate α-syn aggregates. Bar shows 100 μm. Graph shows quantitative analysis of striatal aggregates containing Ser129-phosphorylated α-syn or total α-syn. Data represent means ± SD and *P* values were estimated by one-way ANOVA with Bonferroni correction (*, *P* < 0.05; **, *P* < 0.01)

To determine whether Ser129-phosphorylation affects the formation of α-syn aggregates in vivo, we quantified the number of α-syn aggregates in the striatum of the rat AAV-mediated α-syn overexpression model. These samples were obtained from our previous study [18]. In rats expressing A53T mutant α-syn, the striatal α-syn aggregates were extensively phosphorylated at Ser129 (Fig. 8). The number of Ser129-phosphorylated α-syn-positive aggregates accounted for more than 50% of total α-syn aggregates at 2 and 4 weeks (62.1% in 2 weeks and 55.7% in 4 weeks) after viral injection. Additionally, the number of total α-syn aggregates increased to 265.3 ± 33.4/mm^3^ at 2 weeks (*n* = 5) and 591.9 ± 34.6/mm^3^ at 4 weeks (*P* = 0.001, *n* = 3). Rats expressing A53T plus S129A double-mutant α-syn had 285.8 ± 103.9/mm^3^ α-syn aggregates at 2 weeks (*n* = 4) and 585.4 ± 103.3/mm^3^ at 4 weeks (*n* = 3). There was no significant difference in the number of total α-syn aggregates between rats expressing A53T single mutant and A53T plus S129A double-mutant α-syn (*P* = 1.000). These findings showed that Ser129-phosphorylation had no impact on the accumulation of α-syn aggregates in vivo.

## Discussion

To determine the mechanisms and biological role of Ser129-phosphorylation in α-syn aggregate formation, we first examined the regulation of Ser129-phosphorylation in normal soluble α-syn. Our results showed that α-syn was phosphorylated at Ser129 in proportion to the levels of total α-syn. This phenomenon could be explained by two possibilities: 1) responsible kinases are constitutively active in a substrate-dose dependent manner; or 2) this regulation system is maintained by dephosphorylating or degrading excess amounts of Ser129-phosphorylated α-syn in reaction to total α-syn levels. To further explore these possibilities, we examined how the regulation mechanism of Ser129-phosphorylation was disrupted. Our results showed that the intracellular Ca^2+^ concentration was a key factor in kinase-modulated Ser129-phosphorylation. In support of this, Ser129-phosphorylation required CaM function, which controls a variety of kinases in a Ca^2+^-dependent manner [16, 22]. In mitochondrial complex I inhibition by rotenone or MPP^+^, Ser129-phosphorylation was enhanced through an increased influx of extracellular Ca^2+^. However, the CHX-chase experiments showed that rotenone-induced Ser129-phosphorylated α-syn was targeted to the proteasome pathway at the same rate as normally phosphorylated α-syn. It should be noted that in the present experimental condition, ATP-dependent proteasome activity was not lost by mitochondrial impairment. These findings suggested that proteasomal targeting played a role in suppressively controlling Ser129-phosphorylated α-syn levels. Then, we examined the role of Ser129-phosphorylation in insoluble α-syn accumulation. Chronic treatment with a low concentration of rotenone for 5 days induced small amounts of insoluble α-syn. CHX-chase experiments with MG132 showed that insoluble Ser129-phosphorylated α-syn was targeted to the proteasome pathway. This finding suggested that proteasomal targeting also played a role in suppressing accumulation of insoluble Ser129-phosphorylated α-syn.

**Fig. 9:**
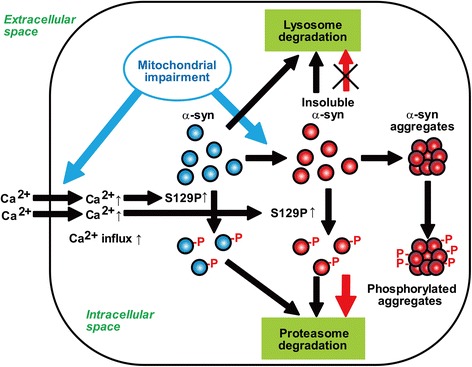
A model of Ser129-phosphorylation role in regulating α-syn levels and forming α-syn aggregates. Mitochondrial impairment stimulates solubility change of α-syn proteins from normally soluble forms to insoluble forms. Also, mitochondrial impairment facilitates Ser129-phosphorylation of α-syn by an increase in influx of extracellular Ca^2+^. Ser129-phosphorylated α-syn, including soluble and insoluble forms, is targeted to the proteasome pathway. Proteasomal targeting of Ser129-phosphorylated α-syn is more promoted under lysosome inhibition. It acts as a suppressor complementary to the lysosome pathway against accumulation of insoluble α-syn proteins. Also, α-syn aggregates undergo Ser129-phosphorylation. However, Ser129-phosphorylation-mediated proteasomal targeting is ineffective, once α-syn aggregates turn to be degradation-resistant. Consequently, α-syn proteins deposited in aggregates are extensively phosphorylated

The present data raised a question of why increased levels of Ser129-phosphorylated α-syn were not accompanied by alteration in the levels of total α-syn in soluble and insoluble forms. To address the issue, we measured the change in levels of Ser129-phosphorylation and total α-syn under proteasome or lysosome inhibition, because α-syn is known to degrade through the autophagy-lysosome pathway [5]. Treatment with chloroquine generated insoluble total α-syn without altering soluble levels, showing that lysosome inhibition preferentially induced the formation of insoluble α-syn proteins. Epoxomicin treatment resulted in no accumulation of soluble or insoluble total α-syn. This could be explained by the hypothesis that Ser129-phosphorylated α-syn, and most non-phosphorylated α-syn proteins, were independently pushed through the proteasome and lysosome pathways, respectively, and the lysosome pathway maintained steady levels of total α-syn under proteasome inhibition. This also supported the finding that chloroquine failed to induce insoluble Ser129-phosphorylated α-syn accumulation, although insoluble total α-syn increased. Additionally, concurrent treatment with epoxomicin and chloroquine increased the higher levels of insoluble Ser129-phosphorylated α-syn than single treatment with epoxomicin or chloroquine. The levels of insoluble total α-syn were more abundant in wild-type α-syn than in S129A α-syn, suggesting that proteasomal targeting of insoluble Ser129-phosphorylated α-syn was promoted to compensate for lysosomal inhibition. This affected insoluble total α-syn accumulation under lysosomal inhibition. We proposed a model for the biological role of Ser129-phosphorylation in the process of α-syn accumulation in Fig. 9.

These findings were consistent with results from a previous yeast study showing that Ser129-phosphorylation and sumoylation push α-syn aggregates into the proteasome pathway and autophagy-lysosome pathway, respectively, and Ser129-phosphorylation rescues autophagy-lysosome clearance of α-syn by promoting proteasomal clearance when sumoylation is impaired [20]. This previous study also showed that Ser129-phosphorylation pushed soluble α-syn monomers into autophagy-lysosomal and proteasome pathways, which was inconsistent with the present results. Our data showed that Ser129-phosphorylation pushed soluble α-syn through the proteasome pathway. This inconsistency could be a result of a difference in yeast and mammalian cell models. Another study reported that PLK2 overexpression selectively induces autophagic clearance of soluble α-syn [14]. However, our results were inconsistent with this finding. We did not overexpress kinases for assessing the effects of Ser129-phosphorylation, which is physiologically mediated by a set of endogenous kinases in cells. Overexpression of each kinase may exert different effects on the degradation of Ser129-phosphorylated α-syn, because PLK2-mediated autophagic clearance of α-syn has also been shown to require binding of PLK2 to α-syn [14].

The present data also raised a question as to relationship between effects of Ser129-phosphorylation on proteasomal targeting of soluble and insoluble α-syn proteins and extensive phosphorylation in α-syn aggregates. To address this, we assessed α-syn aggregates in a rat rAAV model expressing A53T α-syn with or without the S129A mutation. The present data showed that Ser129-phosphorylation had no impact on α-syn aggregate accumulation despite extensive phosphorylation. A previous study demonstrated that fibrillar α-syn proteins were phosphorylated by casein kinase (CK) I or CK II, and they were not good substrates for phosphatases in vitro [24]. Because Ser129-phosphorylation has a role in removing excess amounts of α-syn, α-syn aggregates may continuously undergo phosphorylation. However, it may be ineffective against α-syn proteins, which are degradation-resistant (Fig. 9). Ser129-phosphorylation of α-syn should consider two different aspects: 1) Ser129-phosphorylation may have a protective effect complementary to the lysosome pathway to degrade excess amounts of α-syn; 2) extensive targeting of Ser129-phosphorylated α-syn and degradation-resistant proteins may put a burden on the proteasome pathway. Further studies focused on the relationship between Ser129-phosphorylation and degradation system would provide insight into the potential of modulating the amounts of Ser129-phosphorylation as a new strategy for PD therapy.

## Conclusions

We report that a role of Ser129-phosphorylation in regulating α-syn expression levels is associated with extensive phosphorylation in α-syn aggregates. The levels of Ser129-phosphorylated α-syn were suppressively maintained to be constant to those of total α-syn in intracellular and extracellular spaces. Although mitochondrial impairment by rotenone or MPP^+^ enhanced Ser129-phosphorylation through increased influx of extracellular Ca^2+^, this elevation was suppressively controlled by targeting Ser129-phosphorylated α-syn to the proteasome pathway. This targeting was seen in insoluble α-syn induced by rotenone. Additionally, proteasomal targeting of insoluble Ser129-phosphorylated α-syn was promoted under lysosome inhibition. This complementary action prevented accumulation of insoluble total α-syn. However, in a rat AAV-mediated α-syn overexpression model, Ser129-phosphorylation did not affect α-syn aggregate formation. Taken together, we propose a model that extensive phosphorylation in α-syn aggregates was consequently generated by an ineffective action of Ser129-phosphorylation for removing degradation-resistant α-syn aggregates. 

## Additional files

Additional file 1: Figure S1. Effects of Ca^2+^ on Ser129-phosphorylation of α-syn in rat primary cortical neurons. Cell lysates (10 μg/lane) were loaded on SDS-PAGE and analyzed by western botting with EP1536Y, Syn-1, or anti-β-actin (AC-15) antibody. a Effect of A23187 concentrations on Ser129-phosphorylation. Primary cortical neurons were treated with A23187 at the indicated concentrations for 8 h. b, c Effect of extracellular Ca^2+^ chelator EGTA (b) or intracellular Ca^2+^ chelator BAPTA-AM (B-AM) (c) on A23187-induced Ser129-phosphorylation. Cells were incubated in media containing 0.25 μM A23187 with the indicated concentrations of EGTA or BAPTA-AM for 8 h. d Effect of CaM inhibitor W-7 on A23187-induced Ser129-phosphorylation. Cells were incubated in media containing 0.25 μM A23187 with the indicated concentrations of W-7 for 8 h. Representative blots are shown. Relative band intensities of Ser129-phosphorylated α-syn and total α-syn were normalized to those of β-actin. Graphs show relative ratios to vehicle control cells. Data represent means ± SD and *P* values were estimated by one-way ANOVA with Bonferroni correction or Welch-ANOVA with Games-Howell post hoc test for unequal-variances (*, *P* < 0.05; **, *P* < 0.01). (TIFF 2872 kb)

Additional file 2: Figure S2. Effects of mitochondria complex I inhibitor rotenone on Ser129-phosphorylation of α-syn in rat primary cortical neurons. Cell lysates (5 μg/lane) were loaded on SDS-PAGE and analyzed by western botting with EP1536Y, Syn-1, or AC-15 antibody. a Effect of rotenone concentrations on Ser129-phosphorylation. Primary cortical neurons were incubated in media containing the indicated concentrations of rotenone for 1 h. Vehicle controls were treated with DMSO at the same final concentration. b, c Effect of intracellular Ca^2+^ chelator BAPTA-AM (b) or extracelluar Ca^2+^ chelator EGTA (c) on rotenone-induced Ser129-phosphorylation. Neurons were incubated in media containing 1 nM rotenone and the indicated concentrations of BAPTA-AM or EGTA for 1 h. d Effect of CaM inhibitor W-7 on rotenone-induced Ser129-phosphorylation. Neurons were incubated in media containing 1 nM rotenone with W-7 at the indicated concentrations for 1 h. Representative blots are shown. Relative band intensities of Ser129-phosphorylated α-syn and total α-syn were normalized to those of β-actin. Graphs show relative ratios to vehicle control cells. Data represent means ± SD and *P* values were estimated by one-way ANOVA with Bonferroni correction or Welch-ANOVA with Games-Howell post hoc test for unequal variances (*, *P* < 0.05; **, *P* < 0.01). (TIFF 4854 kb)
