# Supplementary figures and images for: Mechanisms underlying extensive Ser129-phosphorylation in α-synuclein aggregates
Source: Acta Neuropathol Commun. 2017 Jun 15;5:48. doi: 10.1186/s40478-017-0452-6 (PMC5472914; doi:10.1186/s40478-017-0452-6)

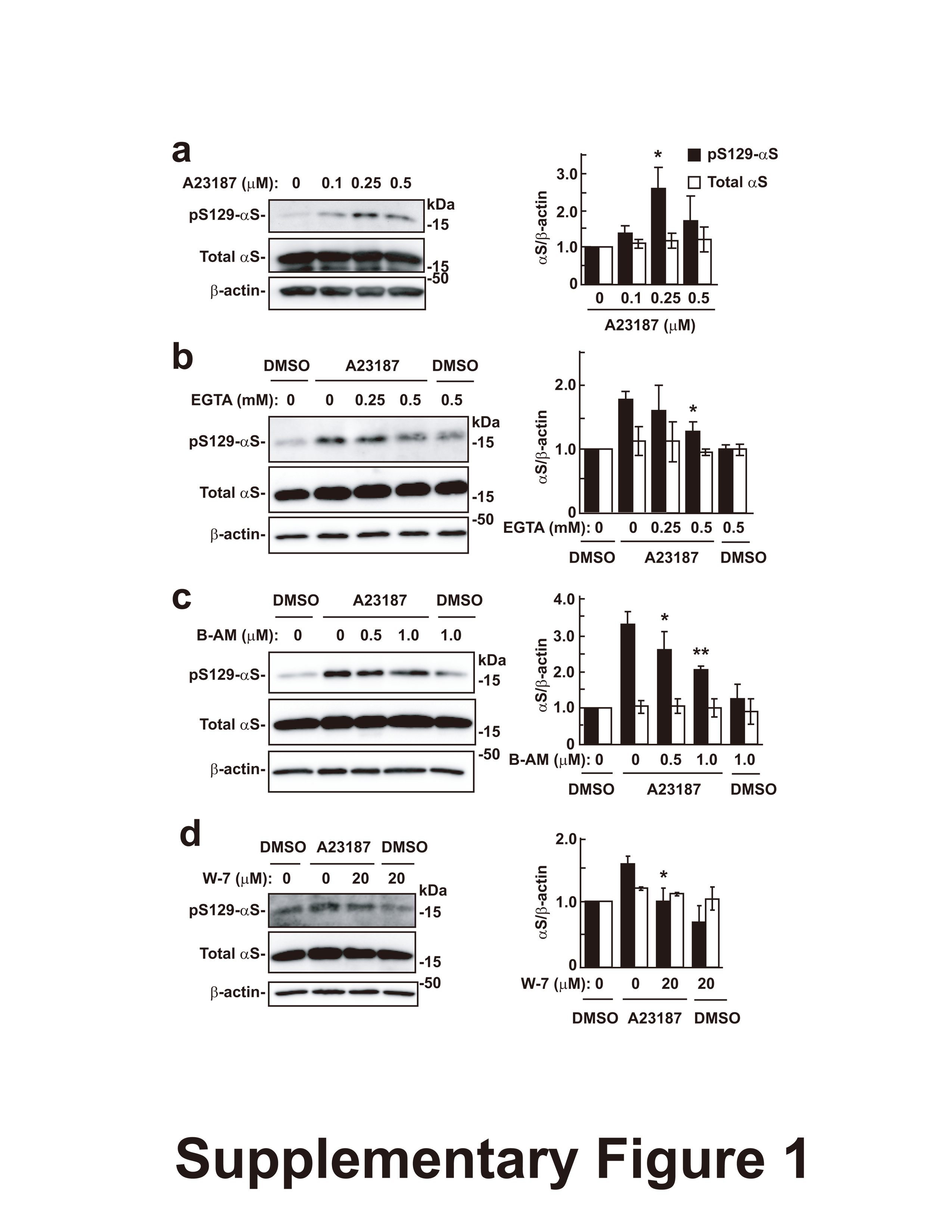

Supplement: Supplementary file 1 — Effects of Ca2+ on Ser129-phosphorylation of α-syn in rat primary cortical neurons. Cell lysates (10 μg/lane) were loaded on SDS-PAGE and analyzed by western botting with EP1536Y, Syn-1, or anti-β-actin (AC-15) antibody. a Effect of A23187 concentrations on Ser129-phosphorylation. Primary cortical neurons were treated with A23187 at the indicated concentrations for 8 h. b, c Effect of extracellular Ca2+ chelator EGTA (b) or intracellular Ca2+ chelator BAPTA-AM (B-AM) (c) on A23187-induced Ser129-phosphorylation. Cells were incubated in media containing 0.25 μM A23187 with the indicated concentrations of EGTA or BAPTA-AM for 8 h. d Effect of CaM inhibitor W-7 on A23187-induced Ser129-phosphorylation. Cells were incubated in media containing 0.25 μM A23187 with the indicated concentrations of W-7 for 8 h. Representative blots are shown. Relative band intensities of Ser129-phosphorylated α-syn and total α-syn were normalized to those of β-actin. Graphs show relative ratios to vehicle control cells. Data represent means ± SD and P values were estimated by one-way ANOVA with Bonferroni correction or Welch-ANOVA with Games-Howell post hoc test for unequal-variances (*, P < 0.05; **, P < 0.01). (TIFF 2872 kb) [file 40478_2017_452_MOESM1_ESM.tif]

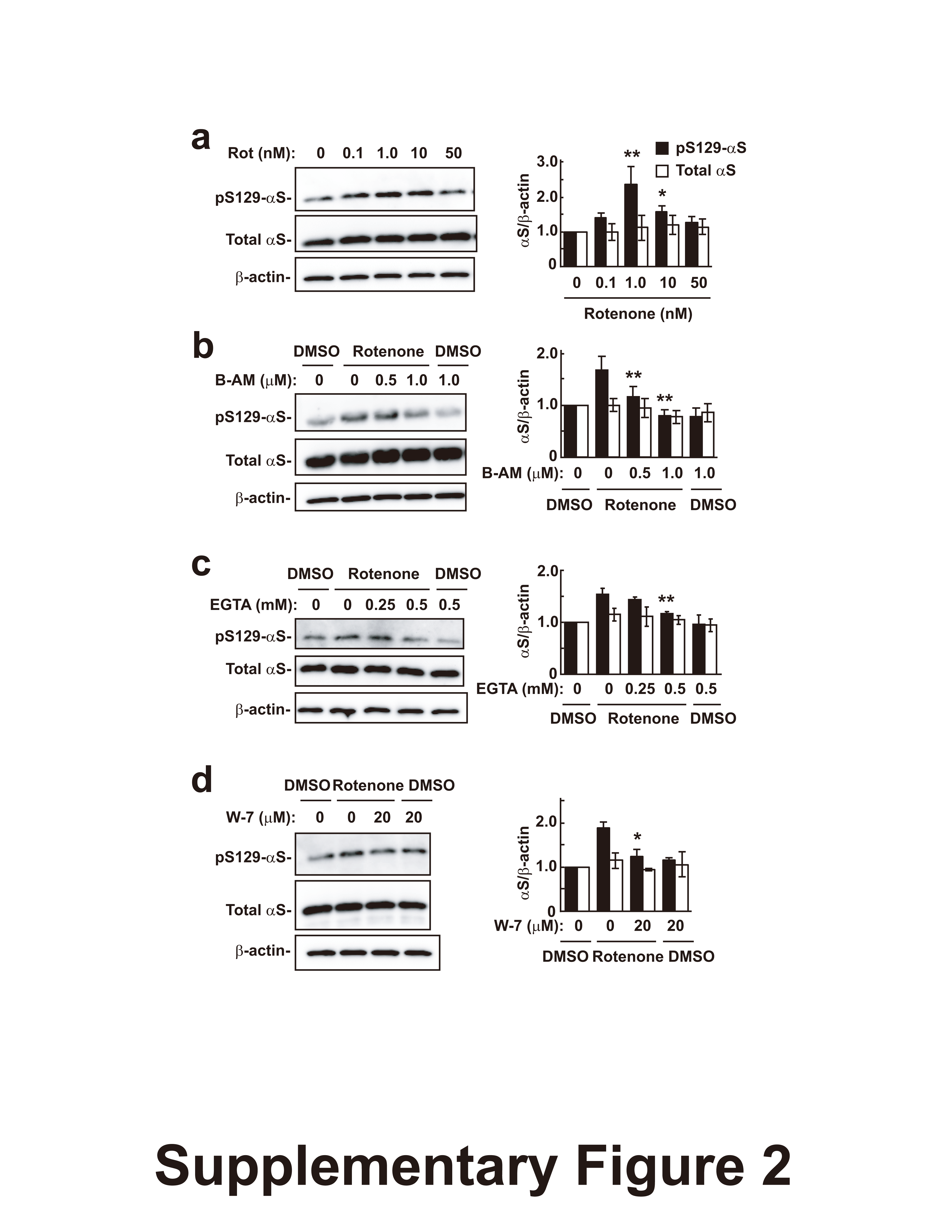

Supplement: Supplementary file 2 — Effects of mitochondria complex I inhibitor rotenone on Ser129-phosphorylation of α-syn in rat primary cortical neurons. Cell lysates (5 μg/lane) were loaded on SDS-PAGE and analyzed by western botting with EP1536Y, Syn-1, or AC-15 antibody. a Effect of rotenone concentrations on Ser129-phosphorylation. Primary cortical neurons were incubated in media containing the indicated concentrations of rotenone for 1 h. Vehicle controls were treated with DMSO at the same final concentration. b, c Effect of intracellular Ca2+ chelator BAPTA-AM (b) or extracelluar Ca2+ chelator EGTA (c) on rotenone-induced Ser129-phosphorylation. Neurons were incubated in media containing 1 nM rotenone and the indicated concentrations of BAPTA-AM or EGTA for 1 h. d Effect of CaM inhibitor W-7 on rotenone-induced Ser129-phosphorylation. Neurons were incubated in media containing 1 nM rotenone with W-7 at the indicated concentrations for 1 h. Representative blots are shown. Relative band intensities of Ser129-phosphorylated α-syn and total α-syn were normalized to those of β-actin. Graphs show relative ratios to vehicle control cells. Data represent means ± SD and P values were estimated by one-way ANOVA with Bonferroni correction or Welch-ANOVA with Games-Howell post hoc test for unequal variances (*, P < 0.05; **, P < 0.01). (TIFF 4854 kb) [file 40478_2017_452_MOESM2_ESM.tif]
